# Usefulness of Transparent Illumination Device in Esophageal Atresia for the Detection of the Distal Esophagus

**DOI:** 10.70352/scrj.cr.25-0803

**Published:** 2026-02-26

**Authors:** Yoshimasa Uematsu, Eiichiro Watanabe, Kiyoshi Tanaka, Hajime Takayasu, Ken-ichiro Konishi, Takuji Tomari, Yusuke Kumamoto

**Affiliations:** Department of General-Pediatric Hepatobiliary Pancreatic Surgery, Kitasato University School of Medicine, Sagamihara, Kanagawa, Japan

**Keywords:** esophageal atresia, optical imaging, pediatrics, surgical procedure, transparent illumination device

## Abstract

**INTRODUCTION:**

Pure esophageal atresia without a tracheoesophageal fistula (Gross type A) is technically demanding, particularly with respect to intraoperative identification of the distal esophageal pouch. Biologically transparent illumination (BTI) devices have been used to visualize nasogastric tubes in pediatric patients; however, their application during esophageal reconstruction has not been reported.

**CASE PRESENTATION:**

A male neonate with prenatally suspected esophageal atresia was diagnosed postnatally with Gross type A esophageal atresia. After gastrostomy and serial bougienage of both esophageal pouches, definitive esophageal reconstruction was performed at 106 days of age. At right thoracotomy, the proximal esophageal pouch was readily identified, whereas the distal pouch was deeply located within the mediastinum and could not be detected visually. A BTI catheter emitting red light from its tip was introduced into the distal esophageal pouch via gastrostomy. Activation of the device allowed clear transillumination through surrounding tissues, enabling accurate localization of the distal esophagus. Subsequent mobilization and end-to-end esophageal anastomosis were successfully completed. Postoperative contrast esophagography showed no evidence of anastomotic leakage or stricture, and enteral feeding with milk was initiated on POD 7. The patient was discharged on POD 24 and has remained free of complications during 10 months of postoperative follow-up.

**CONCLUSIONS:**

This case demonstrates that BTI is a useful device for localizing the distal esophageal pouch during surgery in Gross type A esophageal atresia. The technique may facilitate safer and more reliable esophageal reconstruction in technically challenging pediatric cases.

## Abbreviations

BTI
: biologically transparent illumination
LED
: light emitting diode

## INTRODUCTION

Pure esophageal atresia without a tracheoesophageal fistula, known as Gross type A esophageal atresia, presents significant intraoperative challenges, particularly in identifying the distal esophageal pouch. In many cases, the distal esophagus is deeply buried in the mediastinum and may be difficult to detect at the time of surgical exposure. A device employing BTI has been reported to be effective in pediatric cases for detecting the position of a nasogastric tube in the stomach.^1)^ To our knowledge, the intraoperative application of a BTI device for identifying the distal esophagus in Gross type A esophageal atresia has not been reported. Here, we describe the successful use of a BTI catheter to facilitate intraoperative localization of the distal esophageal pouch.

## CASE PRESENTATION

**Fig. 1:**
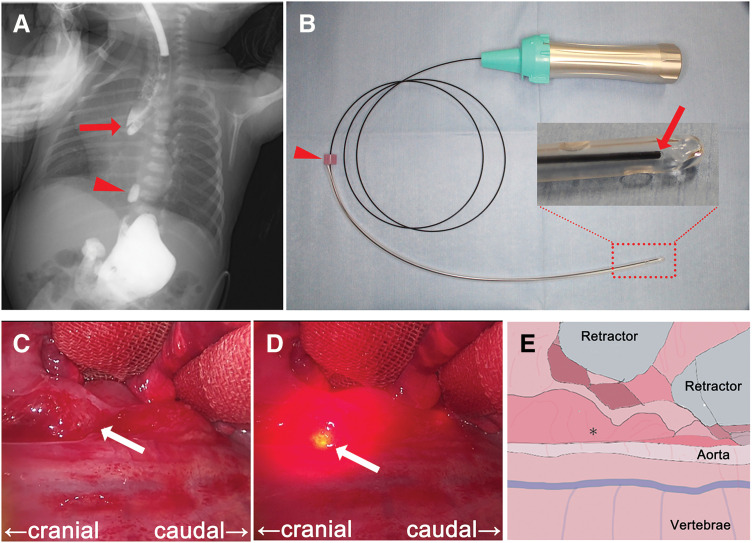
Intraoperative identification of the distal esophageal pouch using biologically transparent illumination. (**A**) Simultaneous contrast study of proximal (red arrow) and distal (red arrowhead) esophagus. The distance between the proximal and distal esophagus spans approximately 3.5 thoracic vertebrae. (**B**) The Tumguide fiber and LED light source. The Tumguide fiber was inserted into a 14 Fr Nelaton catheter and secured with a slide stopper (red arrowhead). The tip (red arrow) illuminated when the light source was activated. (**C**–**E**) Surgical field near the distal esophagus. Through a gastrostomy, a BTI catheter is inserted into the distal esophageal pouch. The position of the distal esophagus pouch is not detectable with the light turned off (**C**) but is visible when the light is turned on (**D**). The brightest point, which represents the catheter tip, indicates the location of the distal esophageal pouch (white arrow). The schematic diagram is shown in (**E**). The distal esophageal pouch is identified at the site indicated by the asterisk (*). BTI, biologically transparent illumination; LED, light emitting diode

A Japanese male infant with prenatally suspected esophageal atresia was delivered by emergency cesarean section due to preterm premature rupture of membranes at 35 weeks of gestation, with a birth weight of 2.2 kg. Postnatal diagnosis was confirmed by the inability to pass a gastric tube and a plain abdominal radiograph showing no gas in the gastrointestinal tract, consistent with Gross type A esophageal atresia. A gastrostomy was performed on day 3 of life. The distance between the proximal and distal esophageal pouches was evaluated using a simultaneous contrast study, revealing a gap equivalent to 3.5 thoracic vertebral bodies (**Fig. 1A**). Serial bougienage of the proximal esophagus was initiated on day 15, followed by bougienage of the distal esophagus on day 35. After 3 months, the gap had decreased to approximately one vertebral body, and definitive esophageal reconstruction was scheduled at 106 days of age, when the patient weighed 4.4 kg.

After induction of general anesthesia, a BTI catheter (Tumguide fiber, 1.0 mm diameter; Otsuka Clinical Solutions, Uruma, Okinawa, Japan), coupled with a light source based on a LED (Tumguide LED Light Source; Neuroceuticals, Tokyo, Japan), was prepared under sterile conditions (**Fig. 1B**). The BTI catheter was inserted into a 14-Fr Nelaton catheter in accordance with the manufacturer’s guidelines. Off-label use of the device was approved by the institutional Ethics Committee. The catheter was secured with a slide stopper and introduced into the distal esophageal pouch via the gastrostomy, and its position was confirmed radiographically. A right thoracotomy was performed through the sixth intercostal space. The proximal esophageal pouch was easily identified near the azygos vein as a dilated structure, whereas the distal esophagus was buried within the mediastinum and initially undetectable. After mobilization of the proximal pouch, exploration of the distal esophagus was undertaken. Upon activation of the BTI device, a red light emitted from the catheter tip was clearly visible within the operative field, enabling accurate localization of the distal esophageal pouch (**Fig. 1C**, **1D**). The distal esophagus was mobilized, and primary end-to-end anastomosis was completed using 5-0 absorbable sutures. The operative time was 319 minutes, with a small amount of blood loss.

The postoperative course was uneventful. Extubation was performed on POD 3. A contrast esophagography on POD 6 showed no evidence of anastomotic leakage or stenosis. Enteral feeding with milk via the gastrostomy tube was initiated on POD 7, and oral milk feeding was added the following day. Full feeding was achieved on POD 11, and the patient was transitioned to complete oral intake on POD 14. As the gastrostomy tube was deemed unnecessary, it was removed on POD 20, and the patient was discharged on POD 24. During long-term follow-up, contrast esophagography at 4 months postoperatively and upper gastrointestinal endoscopy at 6 months postoperatively demonstrated no anastomotic stricture. The patient is currently doing well without complications at 10 months after surgery, and follow-up contrast esophagography is planned every 1–2 years to evaluate for anastomotic stenosis and gastroesophageal reflux.

## DISCUSSION

This case demonstrates the usefulness of a BTI device for detecting tissues that are otherwise difficult to identify intraoperatively. In the search for the distal esophagus in Gross type A esophageal atresia, conventional identification has relied on manipulating a tube placed in the distal esophagus and observing tissue movement within the operative field, as well as on tactile feedback directly perceived by the surgeon. However, in long-gap cases in which the distal esophagus is deeply buried, or in cases accompanied by severe adhesions, identifying the distal esophagus based solely on these methods is often challenging and may carry a risk of injury to surrounding organs. The BTI system delivers light at a wavelength of 660 nm through an optical fiber, and emitted light is most intense perpendicular to the cross-section of the catheter.^2)^ With the addition of optical information provided by BTI, whereby the point of maximum light intensity indicates the blind end of the distal esophagus, it becomes possible to reach the distal esophagus via the shortest route, potentially reducing the risk of injury to adjacent organs.

In previous reports on the use of BTI device during nasogastric tube insertion for detecting the position of the tube in the stomach, the mean or median abdominal wall thickness through which BTI light could be visualized ranged from 9.8 to 21.0 mm, whereas visualization was not possible when the abdominal wall thickness ranged from 20.1 to 30.3 mm.^1–3)^ In addition, reduced visibility of transmitted light has been reported in patients with jaundice or dark skin color.^4)^ These findings suggest that, in cases where the distal esophagus is very deeply buried, or when visualization of transmitted light is impaired by bleeding or mediastinal edema, the applicability of this technique may be limited. However, even when visibility of the transmitted light is reduced, dimming the surrounding illumination has been reported to improve visibility, allowing for practical countermeasures in such situations.^4)^

Similar to endoscopic transillumination, BTI has been applied in several clinical settings, including percutaneous endoscopic gastrostomy, to outline gastric contours and to identify the distal esophagus in cases of esophageal obstruction.^5,6)^ In pediatric practice, BTI devices have also been used to confirm the intragastric position of nasogastric tubes, including in neonates.^1,4,7)^ The present case extends these applications by demonstrating the feasibility of BTI for intraoperative localization of the distal esophagus in Gross type A esophageal atresia.

## CONCLUSIONS

BTI provides a practical visualization of localizing the distal esophageal pouch during surgery in patients with Gross type A esophageal atresia. This technique can be safely applied in pediatric patients and may contribute to more reliable esophageal reconstruction in technically challenging cases.

## DECLARATIONS

### Availability of data and materials

The datasets supporting the conclusions of this article are included within the article.
